# *In silico* evaluation and exploration of antibiotic tuberculosis treatment regimens

**DOI:** 10.1186/s12918-015-0221-8

**Published:** 2015-11-14

**Authors:** Elsje Pienaar, Véronique Dartois, Jennifer J. Linderman, Denise E. Kirschner

**Affiliations:** Department of Chemical Engineering, University of Michigan, 2800 Plymouth Rd, Ann Arbor, MI 48109 USA; Department of Microbiology and Immunology, University of Michigan Medical School, 6730 Medical Science Building II, Ann Arbor, MI 48109 USA; Public Health Research Institute and New Jersey Medical School, Rutgers, The State University of New Jersey, 225 Warren Street, Newark, NJ 07103 USA

**Keywords:** Computational model, Pharmacokinetic/Pharmacodynamic, Isoniazid, Rifampin, Tissue distribution

## Abstract

**Background:**

Improvement in tuberculosis treatment regimens requires selection of antibiotics and dosing schedules from a large design space of possibilities. Incomplete knowledge of antibiotic and host immune dynamics in tuberculosis granulomas impacts clinical trial design and success, and variations among clinical trials hamper side-by-side comparison of regimens. Our objective is to systematically evaluate the efficacy of isoniazid and rifampin regimens, and identify modifications to these antibiotics that improve treatment outcomes.

**Results:**

We pair a spatio-temporal computational model of host immunity with pharmacokinetic and pharmacodynamic data on isoniazid and rifampin. The model is calibrated to plasma pharmacokinetic and granuloma bacterial load data from non-human primate models of tuberculosis and to tissue and granuloma measurements of isoniazid and rifampin in rabbit granulomas. We predict the efficacy of regimens containing different doses and frequencies of isoniazid and rifampin. We predict impacts of pharmacokinetic/pharmacodynamic modifications on antibiotic efficacy. We demonstrate that suboptimal antibiotic concentrations within granulomas lead to poor performance of intermittent regimens compared to daily regimens. Improvements from dose and frequency changes are limited by inherent antibiotic properties, and we propose that changes in intracellular accumulation ratios and antimicrobial activity would lead to the most significant improvements in treatment outcomes. Results suggest that an increased risk of drug resistance in fully intermittent as compared to daily regimens arises from higher bacterial population levels early during treatment.

**Conclusions:**

Our systems pharmacology approach complements efforts to accelerate tuberculosis therapeutic development.

**Electronic supplementary material:**

The online version of this article (doi:10.1186/s12918-015-0221-8) contains supplementary material, which is available to authorized users.

## Background

Pulmonary tuberculosis (TB) results from *Mycobacterium tuberculosis* (Mtb) infection. TB is treatable, but remains a significant public health problem worldwide [1]. Lengthy treatment, requiring at least 6 months of chemotherapy with multiple antibiotics, contributes to patient non-compliance, relapse, drug-resistance, and toxicity, creating an urgent need for shorter regimens and less frequent dosing [2–5]. New approaches are desperately needed for improving TB treatment [6] and include both designing new treatment regimens and developing new antibiotics.

Current regimen design is largely based on efficacy data in mouse models and has not led to dramatic improvements to standard protocols [7]. New regimen design is difficult due to limited understanding of basic mechanisms driving clinical outcomes [7] and because standardized, side-by-side comparisons between treatment regimens are lacking [8–10]. New anti-TB antibiotics are being developed, but it is difficult to predict clinical efficacy based on *in vitro* experiments or even pre-clinical efficacy data as illustrated by disappointing results from recent clinical trials [11–13].

Though it is known that sufficient exposure of bacteria to antibiotics is key to effective TB treatment, dynamics of antibiotics and Mtb at sites of infection, i.e. within granulomas, remain largely unknown and difficult to assess. Granulomas are dense, roughly spherical, complex immunological structures that form upon Mtb infection. Granulomas isolate and control Mtb as well as provide a niche for its persistence. These structures complicate treatment since antibiotic penetration into granulomas is often heterogeneous, and bacteria develop phenotypic tolerance to antibiotics inside granulomas [14].

**Fig. 1:**
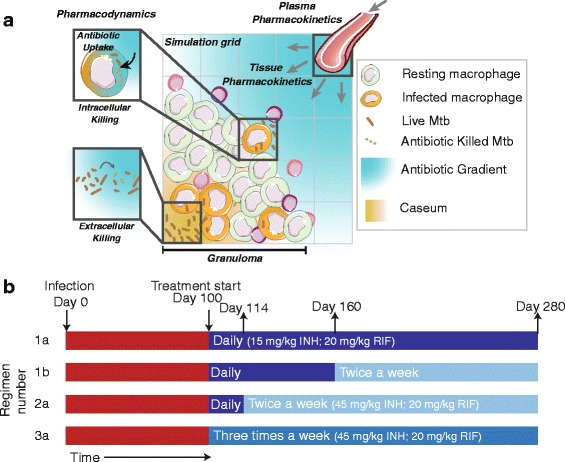
**a** Computational model. Granuloma formation and function, plasma pharmacokinetics (PK), tissue PK and pharmacodynamics (PD) are integrated into a single computational framework. Cell recruitment, movement, states (e.g. activated), actions (e.g. tumor necrosis factor secretion), interactions (e.g. macrophage activation) and death of macrophages and T-cells are followed over time, with granuloma formation and function as emergent behavior. Bacteria are represented as three subpopulations: intracellular, extracellular replicating and extracellular non-replicating (i.e. residing in caseous areas). Plasma PK equations determine the concentration of antibiotic at vascular source sites on the simulation grid. Antibiotics permeate the vascular wall, diffuse within the granuloma, penetrate host cells and kill bacteria based on local intracellular and extracellular concentrations. Further model details are available in [20]. Artwork in (**a**) was constructed by combining and modifying artwork elements from Servier Medical Art (http://www.servier.com/Powerpoint-image-bank) provided under the Creative Commons Unported License 3.0. **b** Simulated antibiotic dosing regimens. Simulated infections are initiated at day 0 and granulomas evolve for the first 100 days (red bars). Regimens 1a, 1b, 2a and 3a, recommended by the CDC/WHO [22], are composed of different doses and frequencies. Regimens 1b and 2a switch from daily to 2 doses per week after 60 and 14 days of treatment, respectively. Each regimen is implemented with INH, RIF and INH + RIF

New antibiotics and regimens must be designed based on a strong pharmacokinetic (PK)-pharmacodynamic (PD) rationale, mindful of the complexities facing antibiotic penetration into and activity within granulomas [2]. Here we show how systems pharmacology can help narrow the design space of new antibiotic regimens and anti-TB antibiotics. We use our established computational model that integrates spatio-temporal dynamics of host immunity (granuloma formation and function [15–19]), PK (in plasma and in lung tissue) and PD [20] (Fig. 1a). Our approach draws from data in animal studies of TB that exhibit human-like pathology [21]. We use this model to generate a repository of *in silico* granulomas and then “treat” these granulomas with isoniazid (INH) and rifampin (RIF), alone and in combination. We compare current regimens in a side-by-side analysis and, unique to using a computational model, we are able to isolate mechanisms of antibiotic penetration and activity that lead granulomas to sterilization. Further, we propose new antibiotic regimens and antibiotic property modifications that could greatly improve TB treatment.

## Results and discussion

### Daily and intermittent regimens are not equivalent in efficacy and intracellular Mtb dominate bacterial populations during treatment with all regimens tested

While the CDC endorses both daily and intermittent (e.g. 2 or 3 doses per week) antibiotic regimens for TB treatment, direct comparisons between these regimens are lacking. Using our computational model, we evaluate treatment of identical granulomas with four CDC-recommended regimens (Fig. 1b) [22] to determine whether intermittent regimens, using higher or equal drug doses, are equivalent to daily regimens with regard to antibiotic penetration into granulomas and bacterial killing. We use doses of INH and RIF that yield human-equivalent exposure in non-human primates (NHPs) [23]. Our computational model is calibrated to NHP data as much as possible; tissue PK characteristics are based on antibiotic penetration in rabbit granulomas [14, 20] as equivalent NHP data are not available. We generate a repository of 500 *in silico* granulomas, simulate treatment according to current CDC regimens (Fig. 1b), and calculate both average antibiotic concentrations and bacterial numbers for each granuloma over time. We distinguish between three bacterial subpopulations – intracellular, extracellular replicating and extracellular non-replicating; the last represents bacteria trapped in caseum, the necrotic center characteristic of TB granulomas [24]. These bacterial subpopulations have differential susceptibilities to INH and RIF [25–27] and we assign each subpopulation a different C_50_ value (concentration where 50 % of maximum efficacy is achieved) [20].

**Fig. 2:**
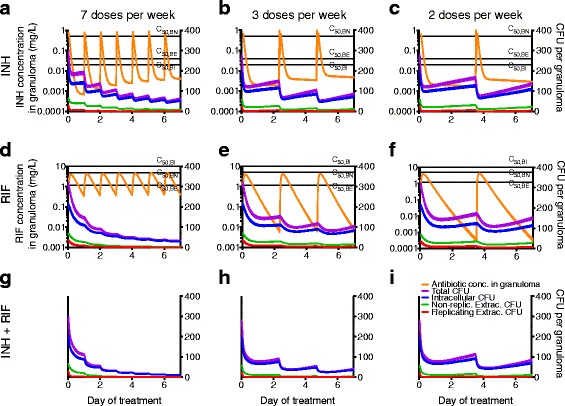
Average bacterial and antibiotic dynamics in simulated granulomas for the first 7 days of treatment with INH (a-c), RIF (d-f) or INH + RIF (g-i) for 7, 3 and 2 doses per week regimens. INH (**a**-**c**) and RIF (**d**-**f**) concentrations (orange curves) inside granulomas are plotted on left vertical-axes; bacterial subpopulations (purple, blue, green and red) are plotted on right vertical-axes (all panels). Solid curves are mean and dashed curves are +/− SEM (*N* = 417). Black lines are C_50_ values (C_50_: concentration where antibiotic reaches 50 % of its maximal activity) for intracellular (C_50,BI_), extracellular (C_50,BE_) and non-replicating (C_50, BN_) bacterial populations

Compared to daily regimens (Fig. 2a, d), intermittent regimens (Fig. 2b, c, e, f) reduce the time during which drug levels remain above C_50_ for both INH and RIF. For both antibiotics we observe bacterial regrowth between doses once antibiotic levels decrease below effective concentrations, and lower dosing frequency leads to increased bacterial growth. For INH, a 7 dose per week regimen shows better efficacy than 3 doses per week despite the larger dose given for 3 doses per week (Fig. 1b). While bacterial regrowth during RIF treatment is less pronounced than during INH treatment, intermittent regimens still allow higher levels of bacteria for RIF and INH + RIF treatment as compared to 7 doses per week. Treating with a combination of INH and RIF lowers bacterial numbers compared to monotherapy for all regimens tested (Fig. 2g-i).

After seven days, nearly all extracellular bacteria are eliminated, independent of antibiotic or regimen (Fig. 2). Elimination of extracellular Mtb is responsible for the steepest decreases in total colony forming units (CFU, a measure of bacterial load). Intracellular Mtb are eliminated more slowly, and continue to dominate total bacterial populations for all regimens during the 6-month treatment period (Additional file 1: Figure S1). The proportions of extracellular non-replicating populations increase once regimens 1b and 2a switch from daily to intermittent phase (Additional file 1: Figure S1).

### Antibiotic exposure within granuloma interiors is below effective levels for all regimens

**Fig. 3:**
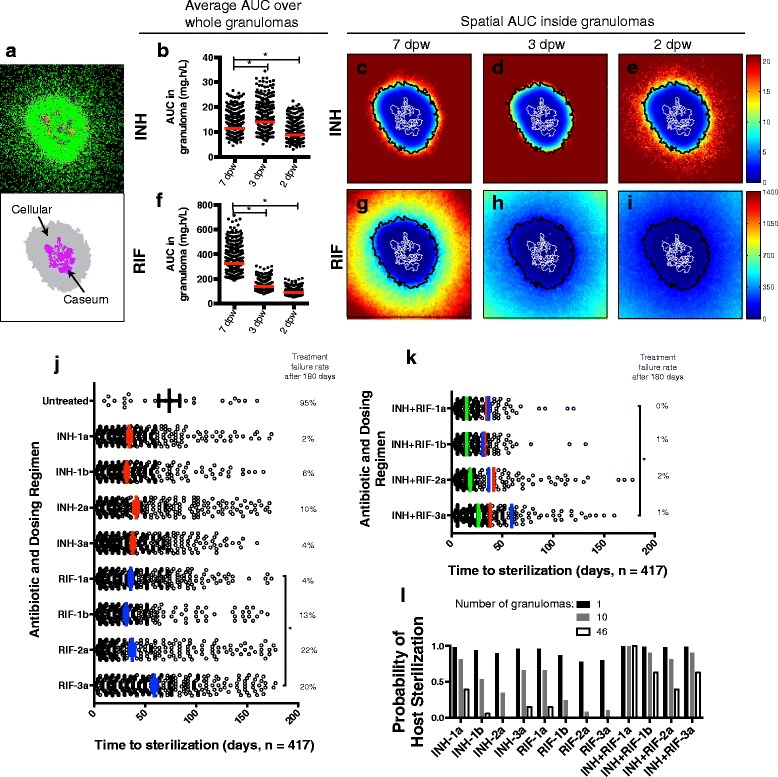
Antibiotic distribution, cumulative exposure (AUC) and treatment outcomes in simulated granulomas. **a** Simulated granuloma snapshot at 100 d.p.i. with cellular (gray) and caseated (purple) areas indicated. Snapshot shows resting (green), activated (blue), and infected (orange and red) macrophages; extracellular bacteria (brown); T cells (purple, pink and light blue). **b** INH 7 day AUC (mg.h/L) averaged over entire granulomas for all simulated granulomas (*N* = 417). **c**-**e** INH 7 day AUC as a function of position in the granuloma in panel (**a**). AUCs are calculated over the first week of treatment with three dosing regimens: 7 doses per week, 3 doses per week, and 2 doses per week. **f**-**i** Same as (**b**-**e**) but for RIF. Cellular and caseum outlines from (**a**) are shown in black and white lines respectively in (**c**-**e**; **g**-**i**). Color bars are scaled from 0 mg.h/L to the AUC EC_80_ (exposure where 80 % of maximal killing is achieved) for each antibiotic. **j** Treatment outcomes for single antibiotic therapy. Each circle represents one granuloma sterilized during 180 days of treatment. Bars and errors bars: mean +/− SEM of time to sterilization. Numbers on the right show treatment failure rates. **k** Treatment outcomes for combination therapy with INH and RIF. Green bars and errors bars: mean +/− SEM. Red and blue lines indicate means for INH (red) and RIF treated granulomas (blue) from (**j**). **l** Probability of a host with 1, 10 or 46 granulomas sterilizing all granulomas during 6 months of treatment. *: *p* < 0.0001 (one way ANOVA with Sidak multiple comparison correction)

Temporal dynamics in antibiotic concentration can be captured by cumulative exposure measures such as area under the concentration curve (AUC), which correlates with efficacy for INH and RIF [28, 29]. Our model results (Fig. 3a) provide first time predictions of AUC averaged over entire granulomas (Fig. 3b, f) as well as for specific locations within granulomas (Fig. 3c-e, g-i) during the first week of treatment. These values have not yet been experimentally determined.

Average INH AUC over entire granulomas is significantly higher for 3 doses per week regimen than for 7 doses per week (Fig. 3b). This increase is due to higher INH doses (45 mg/kg; Fig. 1b) used for intermittent regimens. However, in examining spatial variation of INH AUC inside granulomas (Fig. 3c-e), note that higher average AUC for the 3 doses per week regimen reflects an increase in AUC in the granuloma periphery while interior exposure remains low. Conversely, average and spatial INH exposure within granulomas for the 2 doses per week regimen is lower than for 7 doses per week. For RIF there is a noticeable decrease in average (Fig. 3f) and spatial (Fig. 3g-i) AUC as frequency decreases, since RIF doses are not increased with decreasing frequency (following protocol) [22]. Taken together, these predictions suggest that antibiotic exposure in granuloma interiors (where bacteria reside) is well below effective exposures in all regimens tested.

### Intermittent regimens show higher treatment failure rates and longer times to sterilization than daily regimens; combination therapy improves outcomes

Data connecting antibiotic dynamics to long-term treatment success are lacking and challenging to obtain experimentally. We can probe how drug exposure differences between regimens influence long-term treatment outcomes. At the granuloma scale, we define treatment success as elimination of all bacteria from a granuloma and treatment failure as bacteria remaining after 6-months of treatment. We evaluate outcomes of antibiotic treatment by computing the average time to sterilization (Ts) and the treatment failure rate (Fig. 3j). Ts shows little variation between INH regimens and indicates that the majority of granulomas sterilized with INH regimens 1b and 2a were sterilized during the initial phase of daily dosing. Higher treatment failure rates for INH-2a compared to INH-1b indicate that switching to intermittent dosing too early can hamper sterilization. RIF-3a takes significantly longer to sterilize than RIF-1a. RIF is less efficient than INH in terms of treatment failure rates in all the intermittent regimens (1b, 2a, 3a). Since combination therapy is typical, we also evaluated outcomes of INH + RIF treatment (Fig. 3k), assuming antibiotics act independently. Combination therapy shortens Ts and lowers treatment failure rate for all regimens as compared to individual antibiotic regimens. INH + RIF-3a significantly increases Ts compared to INH + RIF-1a.

Assuming high numbers of independently evolving granulomas (46 in NHPs [30]) and using per-granuloma failure rates (Figs. 3j-k), we can calculate the compound probability of a host being cured, i.e. all granulomas being sterilized. Fig. 3l shows that low treatment failure rates at the granuloma scale could lead to significant risk of treatment failure in hosts with multiple granulomas. These results support the hypothesis that switching from daily to intermittent dosing too early (regimen 2a) can lead to higher rates of treatment failure, and that fully intermittent regimens (regimen 3a) significantly delay granuloma sterilization.

### Small increases in dosing frequency could improve outcomes

**Fig. 4:**
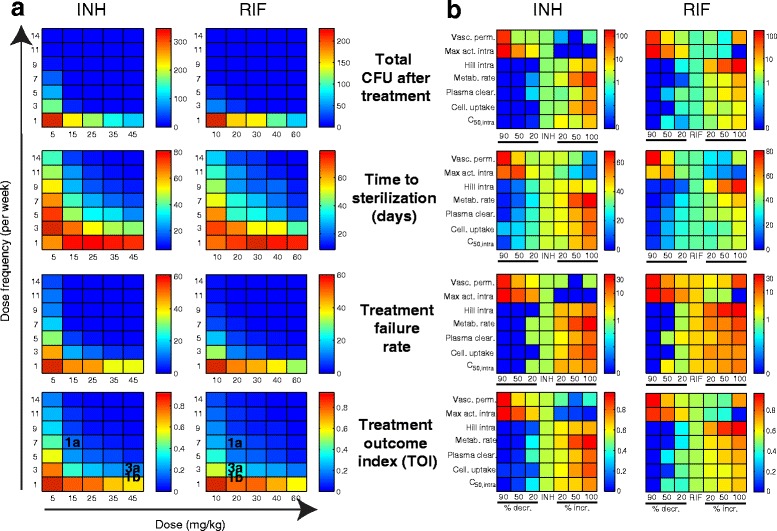
Evaluating antibiotics and regimens improvements. Color scales: means (*N* = 82) for total CFU after treatment, time to sterilization, treatment failure rate and TOI. Red: poor outcomes; blue: good outcomes; dark blue: optimum outcomes. **a** Varying dose (INH: 5–45 mg/kg; RIF: 10–60 mg/kg) and dose frequency (1–14 doses per week). Regimens from Fig. 1b are noted on bottom. **b** Modifying PK-PD properties (vertical axes) previously identified [20] to affect outcomes. Results for INH-1a/RIF-1a (center columns on all panels) are compared with antibiotics where properties were decreased (by 20, 50 or 90 %, left of center) or increased (by 20, 50 or 100 %, right of center) from INH/RIF values. Y-axes show parameters: *vasc*ular *perm*eability; *max*imum killing *act*ivity against *intra*cellular Mtb; *Hill* constant quantifying steepness of dose response killing curve for *intra*cellular Mtb; *rate *of antibiotic *metab*olism inside host cells; rate of antibiotic *clearance* from *plasma*; *Cell*ular *uptake* ratio; *c*oncentration of antibiotic where *50* % of maximum killing activity is achieved against *intra*cellular Mtb

We exploit the power of our systems pharmacology approach to explore INH and RIF doses and frequencies, using multiple treatment outcome metrics (Fig. 4a). As expected, high antibiotic doses at high frequency give the best outcomes in terms of lowering total CFU after treatment, Ts, and treatment failure rate. For both antibiotics, doses above ~15 - 20 mg/kg given 3–5 times a week can achieve significant improvement as measured by total CFU or treatment failure rate. However, a dosing frequency above 7–11 doses per week is needed to minimize Ts.

To provide an overall measure of treatment efficacy, we define a *treatment outcome index, TOI*, a combination of total CFU, Ts and treatment failure rate. INH-1a and RIF-1a perform best by this measure, with improvements predicted if frequencies increase to 9 doses per week or doses to 25 – 30 mg/kg. Toxicity can occur at higher doses and is a concern, especially for INH [31]. Our suggested increases in dose and/or frequency could likely be well-tolerated by patients for RIF [32] but not for INH [33]. To illustrate the trade off between cumulative dose and efficacy: cumulative INH dose for regimen 1a is 105 mg/kg/week. An increase in frequency to 9 doses per week would result in 135 mg/kg/week; an increase in dose only to 25 mg/kg would result in 175 mg/kg/week. Therefore, we predict that increasing INH dose frequency would be a safer option than increasing dose. As evident in our analysis, regimen comparisons will depend on the outcome metrics used. For example, considering CFU after treatment alone, one would conclude that regimens 1a and 3a have similar efficacy. However if one considers the additional outcome metrics or a combination of these outcome metrics (in the form of TOI) it is clear that the two regimens are not equally effective.

### Performance of antibiotics with modified PK and PD properties

Strategic modification of existing antibiotics is an attractive complement to discovery of new compounds. We can evaluate the performances of potential derivatives of INH and RIF by modifying key PK and PD properties and quantifying the extent to which these derivatives are predicted to lower bacterial load, shorten Ts and lower treatment failure rate (Fig. 4b). In previous work, we identified antibiotic properties most influential in determining treatment outcome, including four PK parameters (vascular permeability, plasma clearance rate constant, cellular uptake ratio and drug metabolism rate) and three PD parameters (maximum killing activity, steepness of the dose response killing curve (Hill constant), and C_50_ for intracellular bacteria) [20]. Infection outcome measures for regimens INH-1a and RIF-1a are shown in center columns in all panels of Fig. 4b, and can be compared to other columns within the same panel representing potential new antibiotics with individual PK or PD parameters decreased (by 20, 50 or 90 %) or increased (by 20, 50 or 100 %) from INH or RIF values. As expected, INH and RIF derivatives with increased vascular permeability or maximum intracellular activity show improved outcomes, while the remainder show improved outcomes when corresponding parameters are decreased.

Large changes (>50 %) in antibiotic properties can be difficult to achieve chemically, so we focus our search on antibiotic properties where small changes (10-20 %) from parent drugs are predicted to have a large effect on the outcome of treatment. Total CFU after treatment and treatment failure rate can be minimized by INH derivatives with only a 20 % change in maximum intracellular killing activity, intracellular Hill constant or cellular uptake ratio. RIF derivatives with a minimum of 50 % change in intracellular Hill constant, antibiotic metabolism rate and cellular uptake ratio are required to minimize total CFU after treatment and treatment failure rate. Ts is only minimized by INH and RIF derivatives with 50-90 % changes in properties such as maximum intracellular killing activity and drug metabolism rate. Therefore, derivatives with smaller changes in combinations of these properties might be required to significantly shorten Ts. This approach provides a first-time quantitative guide to strategic antibiotic design by systematically evaluating the benefits of antibiotic modifications.

### Intracellular bacteria present the main reservoir of bacteria at risk of resistance

Clinical observations show that fully intermittent regimens (such as 3a) have an increased risk of selecting for drug resistance [34]. This could be due to sub-therapeutic antibiotic exposure in these regimens (Fig. 3a-i) and/or to larger surviving bacterial populations (Fig. 2) that simply increase the probability of drug resistance. While capturing the development of bacterial resistance is beyond the scope of this study, we are able to identify regions within a granuloma for which antibiotic exposures could lead to increased selection of resistant strains. We perform this analysis for RIF only, building on data from *in vitro* RIF resistance studies that link RIF-resistance to a specific range (0.009 to 0.07) of *C*_*max*_*/C*_*50*_ values [28] (*C*_*max*_: peak concentration during the dosing period); comparable data for INH is not available.

**Fig. 5:**
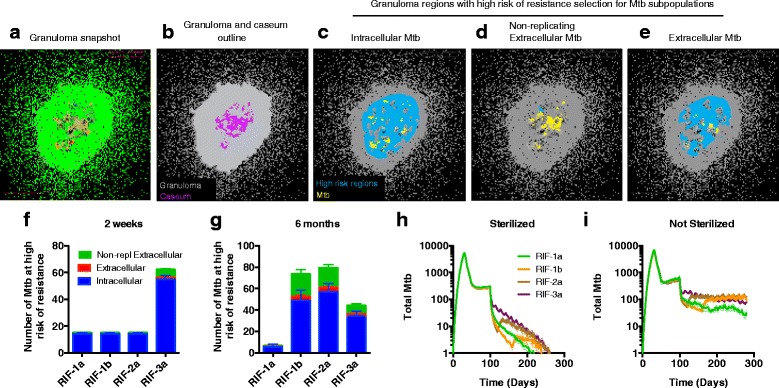
Regions of granuloma predicted to harbor bacterial populations at risk of developing drug resistance during treatment with RIF, and bacterial loads in these high-risk regions. **a** Snapshot of *in silico* granuloma at 100 d.p.i, prior to treatment. **b** Outlines of granuloma (gray) and caseum (purple) for the granuloma in (**a**). **c**-**e** Regions with RIF exposure (*C*_*max*_ */C*_*50*_) in the high-risk range (0.009 to 0.07) are shown in blue for intracellular, non-replicating, and replicating extracellular bacteria. Bacteria in each subpopulation are shown in yellow. **f**, **g** Number of bacteria in high-risk regions after two weeks and 6 months of treatment for all granulomas (*N* = 417). **h**, **i** Total number of Mtb per granuloma, separated based on sterilization. Panels in (**f**-**i**) show mean +/− SEM

We calculate RIF *C*_*max*_*/C*_*50*_ for specific locations in *in silico* granulomas (Fig. 5a, b), and identify regions in granulomas with RIF exposure in the experimentally determined ‘high-risk’ range for each bacterial subpopulation (Fig. 5c-e). The high-risk region for intracellular bacteria (Fig. 5c) is larger than those for non-replicating extracellular (Fig. 5d) and replicating extracellular bacteria (Fig. 5e). However, the development of resistance also depends on how many bacteria reside in these high-risk regions. We quantify the number of bacteria in high-risk regions in all granulomas (Fig. 5f, g) and numbers are reflective of total bacterial load (Fig. 5h, i). At-risk bacterial populations are higher for RIF-3a early on and for RIF-1b and RIF-2a after switching from daily to 2 doses per week, as compared to RIF-1a. For all regimens, the intracellular subpopulation is at highest risk of developing resistance, due to both high proportions of intracellular Mtb (Additional file 1: Figure S1), as well as larger high-risk regions (Fig. 5c). This indicates that the clinically observed increased risk of drug resistance for RIF-3a (compared to RIF-1a) [34] is due to higher intracellular bacterial load in high-risk regions early in treatment.

## Conclusions

TB remains one of the leading infectious diseases worldwide [35]. Experimental studies in appropriate animal models are costly and difficult, and computational models are ideal for generating and testing hypotheses. We evaluate TB treatment using our computational model that integrates host immune dynamics, plasma PK, tissue PK, PD and bacterial dynamics and is calibrated to animal models of TB. The model is well-suited to study TB treatment in the complex granuloma environment, which is difficult to accomplish clinically or in the laboratory.

There is clinical evidence for improved patient compliance and effective treatment of active TB with intermittent regimens [36]. However, direct efficacy comparisons between daily and intermittent regimens are scarce, and existing reports do not agree on the clinical differences between daily and intermittent regimens [34, 37, 38]. Our simulated granulomas do not suffer from cohort-to-cohort variability, which often limits comparison of clinical trials. Our side-by-side comparison of regimens indicates that intermittent INH and RIF regimens have an increased risk of treatment failure and prolonged time to sterilization as compared to daily regimens. We can use the computational model to quickly and systematically test a large number of regimens and quantify improvements that are achievable, acknowledging two major obstacles: drug toxicity associated with increased cumulative exposure to antibiotics, and patient compliance and implementation issues associated with directly observed treatment. While more than double the current dose of RIF is well tolerated in most patients [32, 39], increases in INH exposure are only well-tolerated in patients with fast INH plasma clearance rates [33]. It is increasingly appreciated that antibiotic levels may have an important impact on TB treatment outcomes and emergence of drug resistance [40, 41]. This is particularly true for the rifamycins, as demonstrated in multiple clinical studies of ‘high-dose’ RIF or rifapentine [32, 42]. Increasing dosing frequency can aggravate patient compliance issues, unless an increase in dosing frequency can lead to a significant reduction in treatment duration. Implementation of higher dosing frequencies places increased strain on patients that need to have their dosing observed. However, such stresses could be alleviated by training home supervisors [43] or community members [44] to facilitate DOT. Ultimately, TB treatment is an optimization challenge spanning multiple biological, social and epidemiological scales.

Furthermore, is it more desirable to improve drug penetration or potency? We have shown that antibiotic efficacy is the result of a complex interplay between a variety of PK and PD properties including effective concentrations (C_50_), tissue distribution and drug uptake by host cells, and we have quantified the impact of each property. Our results confirm that over-reliance on potency rather than PK and PD at the site of infection should be avoided [2]. Antibiotic limitations are functions of physico-chemical properties that can be dialed-in by medicinal chemistry, to some extent [45]. For example, QSAR-based design has made significant strides toward rational design of INH derivatives [46, 47], but is almost exclusively focused on lowering MIC [46–48]. A systematic evaluation of the penetration of new drug candidates to the site of action most relevant for their sterilizing activity will undoubtedly help shorten TB treatment and minimize the number of agents required to achieve a sterile cure [45]. We move toward such systematic evaluation by predicting where INH and RIF are limited in the path from blood to lesion to bacterium [2]. Our approach can provide structure and direction to medicinal chemistry efforts and early drug discovery programs.

How antibiotic and bacterial dynamics in the granuloma contribute to the global rise in antibiotic resistant TB, remains unclear. There is a knowledge gap between *in vitro* studies of drug-resistance and clinical observations. Using our model, we offer the first time ability to predict both spatial and temporal risks of resistance in granulomas for different regimens, and can move toward designing desperately needed treatment regimens minimizing drug resistance. *In vivo* studies of drug resistance in NHP models of TB are difficult due to low bacterial numbers per granuloma. *In vitro* resistance studies incorporating PK [28] could be integrated into a next-generation model, shedding light on the contribution of unintentional monotherapy (e.g. in non-replicating bacterial populations where INH performs poorly) to drug resistance development. TB treatment could move beyond traditional antibiotic treatment to include immune-modulation and targeted drug delivery [49, 50], and we have begun to explore those possibilities with our computational model [18, 51].

Our computational approach, particularly when expanded to include additional antibiotics such as bedaquiline, can form part of the international collaborative effort to determine which antibiotics and combinations should be advanced to phase 2 and 3 clinical trials [10] and inform strategies to include immune-modulators in TB treatment [49]. As efforts to shorten treatment regimens beyond the current 6 months have failed in clinical trial stage [7, 11–13], our method provides a straightforward way to understand reasons for inferiority of shorter regimens [7], and to explore new combinations and strategies *in silico*, increasing the probability of clinical trial success.

## Methods

### Agent-based model of antibiotic treatment in TB granulomas

Our next-generation computational model captures granuloma formation and function [16, 17, 19], plasma and lung tissue pharmacokinetics (PK) and pharmacodynamics (PD) of INH and RIF [20] (Fig. 1a). Briefly, our computational agent-based model (ABM) of granuloma formation spans molecular, cellular and tissue scales. The tissue scale comprises cellular movement in chemokine gradients on a 2 dimensional grid. The cellular scale comprises discrete macrophage and T cell agents and their interactions, with cell-specific states (resting, activated, infected or chronically infected for macrophages; and cytotoxic T cells, regulatory T cells or IFN-γ producing T cells). When the cumulative number of host cell deaths in a grid compartment reaches a threshold the compartment becomes caseated. The molecular scale comprises secretion, diffusion, binding and degradation of cytokines (tumor necrosis factor-α, interleukin-10) and chemokines (CCL-2, CCL-5, CXCL-9). Vasculature on the simulation grid is represented by designating a randomly-distributed number of micro-compartments as vascular source compartments where recruited host cells and antibiotics enter the grid. Vascular sources inside the granuloma are considered inactive to reflect lack of vascularization observed *in vivo* [52]. Bacterial populations are captured by continuous variables representing extracellular replicating Mtb (in each ‘healthy’ grid compartment), extracellular non-replicating Mtb (in each caseated grid compartment) and intracellular Mtb (in each macrophage). Bacterial dynamics comprise growth, killing by activated macrophages, killing when infected host cells Mtb reside in undergo apoptosis or cytotoxic killing. Macrophages burst when intracellular Mtb reach the carrying capacity of a macrophage, distributing the bacteria to surrounding grid compartments. Full methodological details are available online (malthus.micro.med.umich.edu/GranSim/).

Antibiotic plasma PK are captured by a four-compartment ODE model of oral antibiotic absorption [14, 20]. Antibiotic tissue PK comprises diffusion and degradation on the simulation grid and penetration into and metabolism by host cells. We implement diffusion as described in [16, 18, 53] using insulating boundary conditions for antibiotic diffusion under the assumption of similar vascularization on grids adjacent to the one under investigation. Cellular accumulation of antibiotics is modeled at pseudo-steady state [54], using a partition coefficient to calculate intracellular and extracellular antibiotic concentrations following diffusion. Antibiotics are added to or subtracted from the vascular source compartments on the grid based on concentration differences between plasma and grid concentrations.

Antibiotic PD are calculated using an *E*_*max*_ model as in [55] with parameters defined separately for intra- or extracellular populations since PD differ between these populations [25, 26]. PD are calculated for each grid compartment or macrophage using local antibiotic concentrations. Antibiotic killing is implemented by subtracting the killing rate (calculated from the *E*_*max*_ model) from the growth rate for each Mtb population.

This model is calibrated to data from NHPs (cynomolgus macaques), the animal model that most closely reproduces human disease and pathology [21], and is continuously curated to incorporate new data. Plasma PK and PD parameters are calibrated to NHP data [23, 30] while tissue PK parameters are calibrated to rabbit data [14] due to a lack of data in NHPs. We assumed similar INH and RIF tissue distribution parameters in rabbits and NHPs. Spatial resolution of INH and RIF concentrations within granulomas is not available from experiments, but can be determined from the computational model. We used C_50_ values from *in vitro* experimental data sets [25, 26] and calibrated other potency parameters to match per granuloma outcomes in INH and RIF treated NHPs [20, 30]. For this work, we modified the computational model to allow us to study INH + RIF combination therapy and multiple dosing regimens. We assumed that INH and RIF are indifferent in combination [56, 57]. Parameter values for host immune functions as well as antibiotic PK and PD are given in Additional file 1: Tables S1 and S2.

### Treatment regimens

We simulated INH- and RIF-containing regimens recommended by the CDC/WHO [22] (Fig. 1b). INH dose size was increased as dosing frequency decreases, but RIF dose size was kept constant even when frequency changes as per protocol. [22] We use doses of INH and RIF that achieve human-equivalent exposure in NHPs [23]. RIF doses are 20 mg/kg, and INH doses are 15 mg/kg for daily dosing and 45 mg/kg for 2 and 3 doses per week.

### Treatment outcome measures

Clinical trials use multiple measures of treatment efficacy [3]. These outcomes are observed in patients, while our focus is on a single granuloma. At the granuloma scale, we define treatment success as elimination of all bacteria from a granuloma (sterilization), and treatment failure as bacteria remaining after treatment. We evaluated treatment outcome based on (i) CFU after treatment (in non-sterilized granulomas), (ii) time to sterilization Ts (in sterilized granulomas) and (iii) the treatment failure rate (percentage of granulomas not sterilized by the end of the treatment period). For comparing regimens, we defined a *treatment outcome index* (TOI): *TOI* = (*w*_*1*_CFU^*^ *+ w*_*2*_Ts^*^ *+ w*_*3*_%Failure^*^)/3; where *w*_*1*_ = *w*_*2*_ = *w*_*3*_ are weights assigned to each outcome measure (i-iii). We assume equal weights. Asterisks indicate normalization between minimum and maximum over all regimens compared. The TOI ranges between 0 and 1, lower values representing “better” outcomes.

### In silico treatment

We generated a repository of 500 *in silico* granulomas, capturing inter-patient variability on a granuloma scale by varying host immune and PK parameters within ranges given in Additional file 1: Tables S1 and S2. We allowed granulomas to evolve for 100 days post infection (d.p.i) before starting treatment. Eighty-three granulomas that cleared infection due to immune action prior to treatment start (at 100 d.p.i) were excluded from the analysis, leaving 417 granulomas in the final set.

For evaluating antibiotic dose, frequency and PK/PD property variations (Fig. 4) we use a separate repository of 82 granulomas that did not sterilize due to immune action prior to treatment start at 100 d.p.i. For dose-frequency variations, INH doses varied between 5 and 45 mg/kg and RIF between 10 and 60 mg/kg. Frequency was varied between 1 and 14 doses per week for INH and RIF. All granulomas were treated with each dose-frequency combination and treatment outcome measures (CFU after treatment, Ts, and treatment failure rate) were calculated as described above.

To explore efficacy of INH and RIF derivatives with different PK and PD properties, the repository of 82 granulomas were treated with regimen INH-1a and RIF-1a using baseline INH and RIF properties [20], or with one of seven key properties varied individually. We generated new antibiotics by decreasing (by 20, 50 or 90 %) or increasing (by 20, 50 or 100 %) individual properties from baseline INH and RIF values. Granulomas were treated with the modified antibiotics and the treatment outcome measures were calculated as described above.

### Risk of drug resistance

*In vitro* experiments in hollow-fiber systems [28] have shown that RIF resistance is amplified in a range of C_max_/MIC. We converted this range to C_max_/C_50_ (C_50_: concentration where antibiotic reaches 50 % of its maximal activity) and calculated this value for each location in the simulated granuloma at the end of each dosing period, enabling us to predict which regions within granulomas are prone to resistance development during a particular dosing regimen. Since equal RIF doses are given for all CDC regimens [22] (Fig. 1b), *C*_*max*_*/C*_*50*_ values are nearly identical for all dosing frequencies (Fig. 2d-f). Therefore, the initial high-risk regions will be similar for all dosing frequencies and we only considered daily dosing. We also quantified the bacteria in each subpopulation in the resistance-prone regions over the entire treatment period. We only performed this analysis for RIF due to the lack of data for INH. INH resistance develops at nearly all concentrations/exposures *in vitro* [27, 29] and *in vitro* resistance mechanisms do not reflect resistance mechanisms seen in patients [58].

## Additional file

Additional file 1: Figure S1. Proportions of bacterial subpopulations (intracellular, replicating extracellular and non-replicating extracellular) before and during treatment with multiple regimens. **Table S1**: Granuloma host parameter baseline values and ranges used to capture patient-to-patient variability in the repository of 500 *in silico* granulomas. **Table S2**: Antibiotic pharmacokinetic (PK) and pharmacodynamic (PD) parameters. (DOCX 855 kb)

## Abbreviations

TB: Tuberculosis
Mtb: *Mycobacterium tuberculosis*
PK: Pharmacokinetics
PD: Pharmacodynamics
INH: Isoniazid
RIF: Rifampin
NHP: Non-human primate
AUC: Area under the concentration curve
Ts: Time to sterilization
TOI: Treatment outcome index
d.p.i: Days post infection
CFU: Colony forming units

## References

[1] WHO. Global tuberculosis report 2014.

[2] Dartois V (2014). The path of anti-tuberculosis drugs: from blood to lesions to mycobacterial cells. Nat Rev Microbiol.

[3] Chang KC, Leung CC, Grosset J, Yew WW (2011). Treatment of tuberculosis and optimal dosing schedules. Thorax.

[4] Zumla A, Nahid P, Cole ST (2013). Advances in the development of new tuberculosis drugs and treatment regimens. Nat Rev Drug Discov.

[5] Saltini C (2006). Schedule or dosage? The need to perfect intermittent regimens for tuberculosis. Am J Respir Crit Care Med.

[6] Nimmo C, Lipman M, Phillips PP, McHugh T, Nunn A, Abubakar I (2015). Shortening treatment of tuberculosis: lessons from fluoroquinolone trials. Lancet Infect Dis.

[7] Warner DF, Mizrahi V (2014). Shortening treatment for tuberculosis--to basics. N Engl J Med.

[8] Menzies D, Benedetti A, Paydar A, Royce S, Madhukar P, Burman W (2009). Standardized treatment of active tuberculosis in patients with previous treatment and/or with mono-resistance to isoniazid: a systematic review and meta-analysis. PLoS Med.

[9] Bose A, Kalita S, Rose W, Tharyan P (2014). Intermittent versus daily therapy for treating tuberculosis in children. Cochrane Database Syst Rev.

[10] Zumla AI, Gillespie SH, Hoelscher M, Philips PP, Cole ST, Abubakar I (2014). New antituberculosis drugs, regimens, and adjunct therapies: needs, advances, and future prospects. Lancet Infect Dis.

[11] Merle CS, Fielding K, Sow OB, Gninafon M, Lo MB, Mthiyane T (2014). A four-month gatifloxacin-containing regimen for treating tuberculosis. N Engl J Med.

[12] Jindani A, Harrison TS, Nunn AJ, Phillips PP, Churchyard GJ, Charalambous S (2014). High-dose rifapentine with moxifloxacin for pulmonary tuberculosis. N Engl J Med.

[13] Gillespie SH, Crook AM, McHugh TD, Mendel CM, Meredith SK, Murray SR (2014). Four-month moxifloxacin-based regimens for drug-sensitive tuberculosis. N Engl J Med.

[14] Kjellsson MC, Via LE, Goh A, Weiner D, Low KM, Kern S (2012). Pharmacokinetic evaluation of the penetration of antituberculosis agents in rabbit pulmonary lesions. Antimicrob Agents Chemother.

[15] Cilfone NA, Ford CB, Marino S, Mattila JT, Gideon HP, Flynn J (2015). Computational modeling predicts interleukin-10 control of lesion sterilization by balancing early host-immunity-mediated antimicrobial responses with caseation during mycobacterium tuberculosis infection. J Immunol.

[16] Cilfone NA, Perry CR, Kirschner DE, Linderman JJ (2013). Multi-scale modeling predicts a balance of tumor necrosis factor-alpha and interleukin-10 controls the granuloma environment during Mycobacterium tuberculosis infection. PLoS One.

[17] Fallahi-Sichani M, El-Kebir M, Marino S, Kirschner DE, Linderman JJ (2011). Multiscale computational modeling reveals a critical role for TNF-alpha receptor 1 dynamics in tuberculosis granuloma formation. J Immunol.

[18] Fallahi-Sichani M, Flynn JL, Linderman JJ, Kirschner DE (2012). Differential risk of tuberculosis reactivation among anti-TNF therapies is due to drug binding kinetics and permeability. J Immunol.

[19] Ray JC, Flynn JL, Kirschner DE (2009). Synergy between individual TNF-dependent functions determines granuloma performance for controlling Mycobacterium tuberculosis infection. J Immunol.

[20] Pienaar E, Cilfone NA, Lin PL, Dartois V, Mattila JT, Butler JR (2015). A computational tool integrating host immunity with antibiotic dynamics to study tuberculosis treatment. J Theor Biol.

[21] Lin PL, Rodgers M, Smith L, Bigbee M, Myers A, Bigbee C (2009). Quantitative comparison of active and latent tuberculosis in the cynomolgus macaque model. Infect Immun.

[22] Blumberg HM, Burman WJ, Chaisson RE, Daley CL, Etkind SC, Friedman LN (2003). American Thoracic Society/Centers for Disease Control and Prevention/Infectious Diseases Society of America: treatment of tuberculosis. Am J Respir Crit Care Med.

[23] Lin PL, Dartois V, Johnston PJ, Janssen C, Via L, Goodwin MB (2012). Metronidazole prevents reactivation of latent Mycobacterium tuberculosis infection in macaques. Proc Natl Acad Sci U S A.

[24] Flynn JL, Gideon HP, Mattila JT, Lin PL (2015). Immunology studies in non-human primate models of tuberculosis. Immunol Rev.

[25] Jayaram R, Gaonkar S, Kaur P, Suresh BL, Mahesh BN, Jayashree R (2003). Pharmacokinetics-pharmacodynamics of rifampin in an aerosol infection model of tuberculosis. Antimicrob Agents Chemother.

[26] Jayaram R, Shandil RK, Gaonkar S, Kaur P, Suresh BL, Mahesh BN (2004). Isoniazid pharmacokinetics-pharmacodynamics in an aerosol infection model of tuberculosis. Antimicrob Agents Chemother.

[27] de Steenwinkel JE, de Knegt GJ, ten Kate MT, van Belkum A, Verbrugh HA, Kremer K (2010). Time-kill kinetics of anti-tuberculosis drugs, and emergence of resistance, in relation to metabolic activity of Mycobacterium tuberculosis. J Antimicrob Chemother.

[28] Gumbo T, Louie A, Deziel MR, Liu W, Parsons LM, Salfinger M (2007). Concentration-dependent Mycobacterium tuberculosis killing and prevention of resistance by rifampin. Antimicrob Agents Chemother.

[29] Gumbo T, Louie A, Liu W, Brown D, Ambrose PG, Bhavnani SM (2007). Isoniazid bactericidal activity and resistance emergence: integrating pharmacodynamics and pharmacogenomics to predict efficacy in different ethnic populations. Antimicrob Agents Chemother.

[30] Lin PL, Coleman T, Carney JP, Lopresti BJ, Tomko J, Fillmore D et al. Radiologic responses in cynomolgous macaques for assessing tuberculosis chemotherapy regimens. Antimicrob Agents Chemother. 2013. doi:10.1128/AAC.00277-13. 10.1128/AAC.00277-13 PMC3754323 23796926

[31] Devarbhavi H (2011). Antituberculous drug-induced liver injury: current perspective. Trop Gastroenterol.

[32] Ruslami R, Ganiem AR, Dian S, Apriani L, Achmad TH, van der Ven AJ (2013). Intensified regimen containing rifampicin and moxifloxacin for tuberculous meningitis: an open-label, randomised controlled phase 2 trial. Lancet Infect Dis.

[33] Possuelo LG, Castelan JA, de Brito TC, Ribeiro AW, Cafrune PI, Picon PD (2008). Association of slow N-acetyltransferase 2 profile and anti-TB drug-induced hepatotoxicity in patients from Southern Brazil. Eur J Clin Pharmacol.

[34] Menzies D, Benedetti A, Paydar A, Martin I, Royce S, Pai M (2009). Effect of duration and intermittency of rifampin on tuberculosis treatment outcomes: a systematic review and meta-analysis. PLoS Med.

[35] McFee RB (2013). Update - pathogens of concern. Dis Mon.

[36] Frieden T. Toman’s tuberculosis: case detection, treatment and monitoring: questions and answers. 2nd ed. Geneva, Switzerland: WHO; 2004.

[37] Chang KC, Leung CC, Yew WW, Chan SL, Tam CM (2006). Dosing schedules of 6-month regimens and relapse for pulmonary tuberculosis. Am J Respir Crit Care Med.

[38] Chang KC, Leung CC, Yew WW, Ho SC, Tam CM (2004). A nested case–control study on treatment-related risk factors for early relapse of tuberculosis. Am J Respir Crit Care Med.

[39] Boeree MJ, Diacon AH, Dawson R, Narunsky K, du Bois J, Venter A et al. A Dose Ranging Trial to Optimize the Dose of Rifampin in the Treatment of Tuberculosis. Am J Respir Crit Care Med. 2015. doi:10.1164/rccm.201407-1264OC. 10.1164/rccm.201407-1264OC 25654354

[40] Srivastava S, Pasipanodya JG, Meek C, Leff R, Gumbo T (2011). Multidrug-resistant tuberculosis not due to noncompliance but to between-patient pharmacokinetic variability. J Infect Dis.

[41] Dartois V (2011). Drug forgiveness and interpatient pharmacokinetic variability in tuberculosis. J Infect Dis.

[42] Boeree MJ, Plemper Van Balen G, Aarnoutse RA (2011). High-dose rifampicin: how do we proceed?. Int J Tuberc Lung Dis.

[43] Wright J, Walley J, Philip A, Pushpananthan S, Dlamini E, Newell J (2004). Direct observation of treatment for tuberculosis: a randomized controlled trial of community health workers versus family members. Trop Med Int Health.

[44] Wandwalo E, Makundi E, Hasler T, Morkve O (2006). Acceptability of community and health facility-based directly observed treatment of tuberculosis in Tanzanian urban setting. Health Policy.

[45] Dartois V, Barry CE (2013). A medicinal chemists’ guide to the unique difficulties of lead optimization for tuberculosis. Bioorg Med Chem Lett.

[46] Martins F, Ventura C, Santos S, Viveiros M (2014). QSAR based design of new antitubercular compounds: improved isoniazid derivatives against multidrug-resistant TB. Curr Pharm Des.

[47] Rajkhowa S, Deka RC (2014). DFT based QSAR/QSPR models in the development of novel anti-tuberculosis drugs targeting Mycobacterium tuberculosis. Curr Pharm Des.

[48] Hearn MJ, Cynamon MH (2004). Design and synthesis of antituberculars: preparation and evaluation against Mycobacterium tuberculosis of an isoniazid Schiff base. J Antimicrob Chemother.

[49] Churchyard GJ, Kaplan G, Fallows D, Wallis RS, Onyebujoh P, Rook GA (2009). Advances in immunotherapy for tuberculosis treatment. Clin Chest Med.

[50] Saifullah B, Hussein MZ (2012). Hussein Al Ali SH. Controlled-release approaches towards the chemotherapy of tuberculosis. Int J Nanomedicine.

[51] Cilfone NA, Pienaar E, Thurber GM, Kirschner DE, Linderman JJ. A systems pharmacology approach towards the design of inhaled formulations of rifampicin and isoniazid for treatment of tuberculosis. CPT: Pharmacometrics and systems pharmacology. 2015;In Press. 10.1002/psp4.22 PMC4394619 26225241

[52] Datta M, Via LE, Kamoun WS, Liu C, Chen W, Seano G et al. Anti-vascular endothelial growth factor treatment normalizes tuberculosis granuloma vasculature and improves small molecule delivery. Proc Natl Acad Sci U S A. 2015. doi:10.1073/pnas.1424563112. 10.1073/pnas.1424563112 PMC4330784 25624495

[53] Cilfone NA, Kirschner DE, Linderman JJ (2014). Strategies for efficient numerical implementation of hybrid multi-scale agent-based models to describe biological systems. Cellular Mol Bioeng.

[54] Goutelle S, Bourguignon L, Maire PH, Van Guilder M, Conte JE, Jelliffe RW (2009). Population modeling and Monte Carlo simulation study of the pharmacokinetics and antituberculosis pharmacodynamics of rifampin in lungs. Antimicrob Agents Chemother.

[55] Bouvier d’Yvoire MY, Maire P (1996). Dosage Regimens of Antibacterials. Clin Drug Invest.

[56] Dickinson JM, Aber VR, Mitchison DA (1977). Bactericidal activity of streptomycin, isoniazid, rifampin, ethambutol, and pyrazinamide alone and in combination against Mycobacterium Tuberculosis. Am Rev Respir Dis.

[57] Bhusal Y, Shiohira CM, Yamane N (2005). Determination of in vitro synergy when three antimicrobial agents are combined against Mycobacterium tuberculosis. Int J Antimicrob Agents.

[58] Bergval IL, Schuitema AR, Klatser PR, Anthony RM (2009). Resistant mutants of Mycobacterium tuberculosis selected in vitro do not reflect the in vivo mechanism of isoniazid resistance. J Antimicrob Chemother.

