# Supplementary material for: In silico evaluation and exploration of antibiotic tuberculosis treatment regimens
Source: BMC Syst Biol. 2015 Nov 14;9:79. doi: 10.1186/s12918-015-0221-8 (PMC4650854; doi:10.1186/s12918-015-0221-8)
Supplement: Additional file 1: Figure S1. — Proportions of bacterial subpopulations (intracellular, replicating extracellular and non-replicating extracellular) before and during treatment with multiple regimens. Table S1: Granuloma host parameter baseline values and ranges used to capture patient-to-patient variability in the repository of 500 in silico granulomas. Table S2: Antibiotic pharmacokinetic (PK) and pharmacodynamic (PD) parameters. (DOCX 855 kb) [file 12918_2015_221_MOESM1_ESM.docx]

Supplement to: *In silico* evaluation and exploration of antibiotic tuberculosis treatment regimens

Elsje Pienaar, Véronique Dartois, Jennifer J. Linderman and Denise E. Kirschner

**Supplemental Figures**

**Figure S1:** Simulated bacterial subpopulations before and during antibiotic treatment. Relative proportions of each bacterial subpopulation (intracellular, extracellular and non-replicating extracellular) are plotted for regimens 1a, 1b, 2a and 3a (rows) with INH, RIF or INH+RIF (columns). Lines indicate means +/- SEM (N=417). Panels are labeled with both antibiotic and treatment regimen (as defined in Fig. 1b). Vertical dashed lines indicate the onset of adaptive immunity (day 30) and antibiotic treatment (day 100).

**Supplemental Tables**

**Table S1:** Granuloma host parameter baseline values and ranges used to capture patient-to-patient variability in the repository of 500 *in silico* granulomas. The baseline parameter set was fitted to experimental measurements of per granuloma bacterial loads from the non-human primate model of TB ([1-4](#_ENREF_1)) as described in ([5](#_ENREF_5)). Further details on these parameters and their implementation in the granuloma model can be found online (malthus.micro.med.umich.edu/GranSim/)

|  |  |  | **Ranges for patient-to-patient variability** | |
| --- | --- | --- | --- | --- |
| **Parameter** | **Unit*** | **Baseline Value** | **Min** | **Max** |
| Bacterial carrying capacity of each grid compartment | Bacteria | 115 |  |  |
| Intracellular bacterial growth rate | h^-1^ | 0.027 |  |  |
| Extracellular bacterial growth rate | h^-1^ | 0.015 |  |  |
| Rate of death of bacteria trapped in caseated compartments | h^-1^ | 5.1 |  |  |
| Number of host cell deaths causing caseation |  | 9 | 7 | 11 |
| Time to heal caseation | Days | 10 | 8 | 12 |
| TNF threshold for causing apoptosis | Molecules | 1150 | 920 | 1380 |
| Rate of TNF induced apoptosis | s^-1^ | 1.7x10^-6^ | 1.3x10^-6^ | 2.04x10^-6^ |
| Minimum chemokine concentration allowing chemotaxis | Molecules | 0.47 | 0.4 | 0.6 |
| Maximum chemokine concentration allowing chemotaxis | Molecules | 480 | 380 | 570 |
| Initial macrophage density | Fraction of grid comp. | 0.04 | 0.03 | 0.05 |
| Time steps before a resting macrophage can move | Timesteps | 3 | 2.4 | 3.6 |
| Time steps before an activated macrophage can move | Timesteps | 19 | 15 | 23 |
| Time steps before an infected macrophage can move | Timesteps | 170 | 135 | 200 |
| TNF threshold for activating NFkB | Molecules | 75 | 60 | 90 |
| Rate of TNF induced NFkB activation | s^-1^ | 1.06x10^-5^ | 8.5x10^-6^ | 1.3x10^-5^ |
| Number of bacteria resting macrophage can phagocytose | Bacteria | 1 |  |  |
| Probability of resting macrophage killing bacteria |  | 0.12 | 0.1 | 0.15 |
| Adjustment for killing probability of resting macrophages with NFkB activated |  | 0.2 | 0.15 | 0.25 |
| Number of extracellular bacteria that can activate NFkB | Bacteria | 250 | 200 | 300 |
| Threshold for intracellular bacteria causing chronically infected macrophages | Bacteria | 12 | 10 | 15 |
| Threshold for intracellular bacteria causing macrophage to burst | Bacteria | 23 | 18 | 30 |
| Number of bacteria activated macrophage can phagocytose | Bacteria | 5 | 4 | 6 |
| Probability of an activated macrophage healing a caseated compartment in its Moore neighborhood |  | 0.0055 | 0.0044 | 0.0066 |
| Probability of a T-cell moving to the same compartment as a macrophage |  | 0.046 | 0.035 | 0.055 |
| IFN γ -producing T-cell probability of inducing Fas/FasL mediated apoptosis |  | 0.035 | 0.03 | 0.04 |
| IFN γ -producing T-cell probability of producing TNF |  | 0.045 | 0.04 | 0.05 |
| IFN γ -producing T-cell probability of producing IFN |  | 0.35 | 0.3 | 0.45 |
| Cytotoxic T-cell probability of killing a macrophage |  | 0.009 | 0.007 | 0.010 |
| Cytotoxic T-cell probability of killing a macrophage and all of its intracellular bacteria |  | 0.7 | 0.6 | 0.9 |
| Cytotoxic T-cell probability of producing TNF |  | 0.05 | 0.04 | 0.06 |
| Regulatory T-cell probability of deactivating activated macrophage |  | 0.008 | 0.006 | 0.01 |
| Time before maximum recruitment rates are reached | Timesteps | 980 | 790 | 1180 |
| Macrophage maximal recruitment probability |  | 0.32 | 0.25 | 0.4 |
| Macrophage chemokine recruitment threshold | Molecules | 0.86 | 0.7 | 1 |
| Macrophage TNF recruitment threshold | Molecules | 0.011 | 0.009 | 0.015 |
| Macrophage half sat for TNF recruitment | Molecules | 1.6 | 1.3 | 2 |
| Macrophage half sat for chemokine recruitment | Molecules | 2.2 | 1.8 | 2.6 |
| IFN γ -producing T-cell maximal recruitment probability |  | 0.15 | 0.12 | 0.18 |
| IFN γ -producing T-cell chemokine recruitment threshold | Molecules | 0.07 | 0.06 | 0.09 |
| IFN γ -producing T-cell TNF recruitment threshold | Molecules | 1.3 | 1 | 1.6 |
| IFN γ -producing T-cell half sat for TNF recruitment | Molecules | 1.3 | 1 | 1.6 |
| IFN γ -producing T-cell half sat for chemokine recruitment | Molecules | 2 | 1.5 | 2.5 |
| Cytotoxic T-cell maximal recruitment probability |  | 0.12 | 0.1 | 0.15 |
| Cytotoxic T-cell chemokine recruitment threshold | Molecules | 4.5 | 3.6 | 5.4 |
| Cytotoxic T-cell TNF recruitment threshold | Molecules | 1.3 | 1 | 1.5 |
| Cytotoxic T-cell half sat for TNF recruitment | Molecules | 1.2 | 1 | 1.5 |
| Cytotoxic T-cell half sat for chemokine recruitment | Molecules | 9 | 7 | 10 |
| Regulatory T-cell maximal recruitment probability |  | 0.03 | 0.02 | 0.04 |
| Regulatory T-cell chemokine recruitment threshold | Molecules | 2 | 1.5 | 2.5 |
| Regulatory T-cell TNF recruitment threshold | Molecules | 1.7 | 1.3 | 2 |
| Regulatory T-cell half sat for TNF recruitment | Molecules | 2.2 | 1.8 | 2.7 |
| Regulatory T-cell half sat for chemokine recruitment | Molecules | 1.5 | 1.2 | 1.8 |

*Conversion factor: 10 min/timestep.

**Table S2:** Antibiotic pharmacokinetic (PK) and pharmacodynamic (PD) parameters. Further details on these parameters and their implementation in the granuloma model can be found online (malthus.micro.med.umich.edu/GranSim/) and in ([5](#_ENREF_5))

| **Parameter Name** | **Units** | **INH** | **RIF** | **Reference** |
| --- | --- | --- | --- | --- |
| **Plasma PK parameters ^(1)^** |  |  |  |  |
| Absorption rate constant (*k_a_*) | h^-1^ | 1-5 | 0.2-0.8 | Fit to data in ([2](#_ENREF_2)) guided by values in ([6-8](#_ENREF_6)) |
| Intercompartmental clearance rate constant (*Q*) | L/h/kg | 0.025-0.2 | 0.1-0.7 | Fit to data in ([2](#_ENREF_2)) guided by values in ([6-8](#_ENREF_6)) |
| Plasma volume of distribution (*V_p_*) | L/kg | 0.1-2 | 0.5-1.5 | Fit to data in ([2](#_ENREF_2)) guided by values in ([6-8](#_ENREF_6)) |
| Peripheral volume of distribution (other organs and tissues) (*V_pe_*) | L/kg | 20-40 | 0.1-1 | Fit to data in ([2](#_ENREF_2)) guided by values in ([6-8](#_ENREF_6)) |
| Plasma clearance rate constant (*CL*) | L/h/kg | 0.6-1.8 | 0.25-0.5 | Fit to data in ([2](#_ENREF_2)) guided by values in ([6-8](#_ENREF_6)) |
|  |  |  |  |  |
| **Lung tissue PK parameters** |  |  |  |  |
| Degradation rate constant, extracellular (*K_deg,e_*) | s^-1^ | 5.5*10^-9^ | 7.5*10^-8^ | Fit to data in ([6](#_ENREF_6)) |
| Degradation rate constant, intracellular (*K_deg,i_*) | s^-1^ | 6.4*10^-3^ | 6.7*10^-3^ | Fit to data in ([6](#_ENREF_6)) |
| Effective diffusivity (*D*) | cm^2^/s | 1.1*10^-7^ | 7*10^-7^ | Fit to data in ([6](#_ENREF_6)) guided by values in ([9](#_ENREF_9)) |
| Cellular accumulation ratio ^(2)^ (*a*) | - | 0.35 | 18 | Fit to data in ([6](#_ENREF_6)) guided by values in ([10-13](#_ENREF_10)) |
| Vascular permeability (*p*) | cm/s | 8.4*10^-6^ | 1*10^-5^ | Fit to data in ([6](#_ENREF_6)) guided by values in ([14](#_ENREF_14)) |
| Permeability coefficient (*PC*) | - | 0.25 | 3.3 | ([6](#_ENREF_6)) |
|  |  |  |  |  |
| **PD parameters** |  |  |  |  |
| C50 for intracellular Mtb (*C_50,BI_*) | mg/L | 0.02 | 10 | ([15-18](#_ENREF_15)) |
| C50 for extracellular replicating Mtb (C*_50,BE_*) | mg/L | 0.04 | 1.23 | ([15-18](#_ENREF_15)) |
| C50 for extracellular non-replicating Mtb (*C_50,BN_*) | mg/L | 0.5 | 5 | ([15-18](#_ENREF_15)) |
| Hill constant for intracellular Mtb (*H_BI_*) | - | 1 | 0.48 | ([15-18](#_ENREF_15)) |
| Hill constant for extracellular replicating Mtb (*H_BE_*) | - | 1 | 0.7 | ([15-18](#_ENREF_15)) |
| Hill constant for extracellular non-replicating Mtb (*H_BN_*) | - | 1 | 0.7 | Assumed same as extracellular replicating |
| Max activity intracellular (*E_max,BI_*) | s^1^ | 7.7*10^-5^ | 1.1*10^-4^ | Fit to data in ([1](#_ENREF_1)) guided by values in ([16](#_ENREF_16), [17](#_ENREF_17)) |
| Max activity extracellular (*E_max,BE_*) | s^-1^ | 2.6*10^-4^ | 5*10^-4^ | Fit to data in ([1](#_ENREF_1)) guided by values in ([16](#_ENREF_16), [17](#_ENREF_17)) |

^(1)^: Plasma PK parameters are given a range of values to account for inter-individual variation

^(2)^: Steady state concentration inside macrophages/concentration outside macrophages

**References**

1. Lin PL, Coleman T, Carney JP, Lopresti BJ, Tomko J, Fillmore D, Dartois V, Scanga C, Frye LJ, Janssen C, Klein E, Barry CE, 3rd, Flynn JL. Radiologic responses in cynomolgous macaques for assessing tuberculosis chemotherapy regimens. *Antimicrobial agents and chemotherapy* 2013.

2. Lin PL, Dartois V, Johnston PJ, Janssen C, Via L, Goodwin MB, Klein E, Barry CE, 3rd, Flynn JL. Metronidazole prevents reactivation of latent mycobacterium tuberculosis infection in macaques. *Proceedings of the National Academy of Sciences of the United States of America* 2012;109:14188-14193.

3. Lin PL, Ford CB, Coleman MT, Myers AJ, Gawande R, Ioerger T, Sacchettini J, Fortune SM, Flynn JL. Sterilization of granulomas is common in active and latent tuberculosis despite within-host variability in bacterial killing. *Nature medicine* 2014;20:75-79.

4. Lin PL, Rodgers M, Smith L, Bigbee M, Myers A, Bigbee C, Chiosea I, Capuano SV, Fuhrman C, Klein E, Flynn JL. Quantitative comparison of active and latent tuberculosis in the cynomolgus macaque model. *Infection and immunity* 2009;77:4631-4642.

5. Pienaar E, Cilfone NA, Lin PL, Dartois V, Mattila JT, Butler JR, Flynn JL, Kirschner DE, Linderman JJ. A computational tool integrating host immunity with antibiotic dynamics to study tuberculosis treatment. *J Theor Biol* 2015;367:166-179.

6. Kjellsson MC, Via LE, Goh A, Weiner D, Low KM, Kern S, Pillai G, Barry CE, 3rd, Dartois V. Pharmacokinetic evaluation of the penetration of antituberculosis agents in rabbit pulmonary lesions. *Antimicrobial agents and chemotherapy* 2012;56:446-457.

7. Wilkins JJ, Langdon G, McIlleron H, Pillai G, Smith PJ, Simonsson US. Variability in the population pharmacokinetics of isoniazid in south african tuberculosis patients. *British journal of clinical pharmacology* 2011;72:51-62.

8. Wilkins JJ, Savic RM, Karlsson MO, Langdon G, McIlleron H, Pillai G, Smith PJ, Simonsson US. Population pharmacokinetics of rifampin in pulmonary tuberculosis patients, including a semimechanistic model to describe variable absorption. *Antimicrobial agents and chemotherapy* 2008;52:2138-2148.

9. Pruijn FB, Patel K, Hay MP, Wilson WR, Hicks KO. Prediction of tumour tissue diffusion coefficients of hypoxia-activated prodrugs from physicochemical parameters. *Australian Journal of Chemistry* 2008;61:687-693.

10. Jeena PM, Bishai WR, Pasipanodya JG, Gumbo T. In silico children and the glass mouse model: Clinical trial simulations to identify and individualize optimal isoniazid doses in children with tuberculosis. *Antimicrobial agents and chemotherapy* 2011;55:539-545.

11. Mor N, Simon B, Mezo N, Heifets L. Comparison of activities of rifapentine and rifampin against mycobacterium tuberculosis residing in human macrophages. *Antimicrobial agents and chemotherapy* 1995;39:2073-2077.

12. Forsgren A, Bellahsene A. Antibiotic accumulation in human polymorphonuclear leucocytes and lymphocytes. *Scandinavian journal of infectious diseases Supplementum* 1985;44:16-23.

13. Ziglam HM, Baldwin DR, Daniels I, Andrew JM, Finch RG. Rifampicin concentrations in bronchial mucosa, epithelial lining fluid, alveolar macrophages and serum following a single 600 mg oral dose in patients undergoing fibre-optic bronchoscopy. *The Journal of antimicrobial chemotherapy* 2002;50:1011-1015.

14. Schmidt MM, Wittrup KD. A modeling analysis of the effects of molecular size and binding affinity on tumor targeting. *Molecular cancer therapeutics* 2009;8:2861-2871.

15. de Steenwinkel JE, de Knegt GJ, ten Kate MT, van Belkum A, Verbrugh HA, Kremer K, van Soolingen D, Bakker-Woudenberg IA. Time-kill kinetics of anti-tuberculosis drugs, and emergence of resistance, in relation to metabolic activity of mycobacterium tuberculosis. *The Journal of antimicrobial chemotherapy* 2010;65:2582-2589.

16. Jayaram R, Gaonkar S, Kaur P, Suresh BL, Mahesh BN, Jayashree R, Nandi V, Bharat S, Shandil RK, Kantharaj E, Balasubramanian V. Pharmacokinetics-pharmacodynamics of rifampin in an aerosol infection model of tuberculosis. *Antimicrobial agents and chemotherapy* 2003;47:2118-2124.

17. Jayaram R, Shandil RK, Gaonkar S, Kaur P, Suresh BL, Mahesh BN, Jayashree R, Nandi V, Bharath S, Kantharaj E, Balasubramanian V. Isoniazid pharmacokinetics-pharmacodynamics in an aerosol infection model of tuberculosis. *Antimicrobial agents and chemotherapy* 2004;48:2951-2957.

18. Gumbo T, Louie A, Liu W, Brown D, Ambrose PG, Bhavnani SM, Drusano GL. Isoniazid bactericidal activity and resistance emergence: Integrating pharmacodynamics and pharmacogenomics to predict efficacy in different ethnic populations. *Antimicrobial agents and chemotherapy* 2007;51:2329-2336.
